# Association of higher arterial ketone body ratio (acetoacetate/β-hydroxybutyrate) with relevant nutritional marker in hemodialysis patients

**DOI:** 10.1186/s12882-020-02173-1

**Published:** 2020-11-25

**Authors:** Masaaki Inaba, Yasuro Kumeda, Shinsuke Yamada, Norikazu Toi, Chie Hamai, Koichi Noguchi, Eikichi Yasuda, Yutaka Furumitsu, Masanori Emoto, Yoshiteru Ohno

**Affiliations:** 1 Kidney Center, Ohno Memorial Hospital, 1-26-10, Minami-Horie, Nishi-ku, Osaka, 550-0015 Japan; 2 Dialysis Center, Minami-Osaka Hospital, 1-18-18, Higashi-kagaya, Suminoe-ku, Osaka, 559-0012 Japan; 3 Department of Metabolism, Endocrinology and Molecular Medicine, Osaka City University Graduate School of Medicine, 1-4-3, Asahi-machi, Abeno-ku, Osaka, 545-8585 Japan

**Keywords:** Ketone, Ketone body ratio, Albumin, Uric acid, Hemodialysis

## Abstract

**Background:**

An association of higher levels of β-hydroxybutyrate (β-HB) in serum with greater mortality in hemodialysis (HD) patients has been reported. This study examined the significance of arterial ketone body ratio (AcAc/β-HB), a relevant marker of energy state, in HD patients.

**Methods:**

The levels of arterial AcAc and β-HB, and AcAc/β-HB ratio were determined in 49 HD patients just before undergoing an HD session. Additionally, changes in those levels during the session were examined to investigate their associations with clinical nutritional markers.

**Results:**

Arterial β-HB, but not AcAc, was significantly higher at the baseline in 25 patients with type 2 diabetes mellitus (T2DM) as compared to 24 non-DM patients, with a significant reduction in arterial AcAc/β-HB ratio seen in those with DM. Although the arterial AcAc/β-HB ratio before the HD session was significantly higher in the non-DM group, it did not differ significantly after the session between the groups, indicating a faster rate of β-HB disappearance from circulation in non-DM HD patients during the interdialytic period. Multiple regression analysis, which included age, gender, presence/absence of DM, log HD duration, log β-HB, and log AcAc/β-HB ratio as independent variables, revealed an independent and significant association of log AcAc/ β-HB ratio, but not log β-HB, with serum albumin and uric acid.

**Conclusion:**

We found that a decreased AcAc/β-HB ratio resulting from increased β-HB, but not increased β-HB itself, was a significant factor independently associated with decreased levels of serum albumin and uric acid, known to be related to higher mortality in HD patients. Furthermore, it is possible that higher mortality in DM HD patients can be explained by reduced arterial AcAc/β-HB ratio.

## Background

Patients undergoing hemodialysis (HD) are known to exhibit a significant increase in serum ketone bodies [acetoacetate (AcAc), β-hydroxybutyrate (β-HB)] during a single HD session [1]. Ketone bodies have long been understood as a better fuel source than glucose or fatty acids [2, 3]. However, it was recently reported that higher serum β-HB was independently associated with increased cardiovascular disease (CVD) events and cases of all-cause death in HD patients in Japan [4]. It has been shown that a reduction in arterial ketone body ratio, defined by determining arterial AcAc/β-HB ratio, a non-invasive method for evaluating hepatic energy charge [5], is a novel independent CVD risk factor [6], thus it is important to examine whether higher β-HB by itself or a reduction in arterial AcAc/β-HB ratio resulting from higher β-HB as well function to contribute to worse outcome of HD patients. Furthermore, investigation of the significance of changes in arterial AcAc, β-HB, and AcAc/β-HB ratio occuring during an HD session is of interest. Also, because of the effect of insulin to suppress production of arterial ketone bodies [7], it is possible that stimulation of ketone body production resulting from inhibition of insulin secretion in patients treated with dialysate containing 125 mg/dL glucose might differ between those with type 2 diabetes mellitus (T2DM) and non-DM HD patients.

These background factors prompted us to examine arterial AcAc/β-HB ratio and β-HB to determine which might be better to predict nutritional state, and thus clinical outcome in both T2DM and non-DM HD patients. Additionally, the mechanism of changes in serum β-HB and AcAc together with arterial AcAc/β-HB ratio during an HD session was investigated.

## Methods

### Patients

For the present study, 49 HD patients (25 T2DM, 24 non-DM) receiving four-hour treatments three times per week at the outpatient clinic of Minami-Osaka Hospital Kidney Center, Japan, were enrolled. Written informed consent was obtained from each prior to participation. The protocol was approved by the Ethics Review Committee of Minami-Osaka Hospital (Approval #2015–10) and conducted in accordance with the principles of the Declaration of Helsinki. All patients provided consent to participate in this study. Those who had been undergoing HD therapy for less than 1 year or more than 21 years were excluded, as previously described [8, 9].

### Sample collection

Arterial blood samples were drawn twice from the arteriovenous fistula either before or after a morning HD session at the beginning of the week, 3 days after the previous HD session, and following an overnight fast [10]. Furthermore, to assess changes in arterial blood AcAc, β-HB, and ketone body ratio, defined by arterial AcAc/β-HB ratio, during a single four-hour HD session, blood samples were also obtained just after the session had finished. A portion of the sampled arterial blood was used for measurements of acid-base parameters (pH, bicarbonate). The remaining specimen was kept at 4 °C for 1 h, then centrifuged at 1000 x *g* for 10 min and stored in aliquots at − 80 °C until being assayed. Prior to the assay, the frozen sample was thawed and measurements were performed immediately thereafter.

### Laboratory measurements

Just before and after the HD session, in addition to standard parameters, ketone bodies (AcAc, β-HB) were measured in arterial serum samples obtained from the arteriovenous fistula using commercially available kits. Arterial blood samples obtained simultaneously were measured for acid-base parameters (pH, bicarbonate) using a blood gas analyzer. Arterial ketone body (AcAc/β-HB) ratio was estimated as redox state in liver mitochondria capable of producing ATP [11]. AcAc and β-HB were measured using commercially available calorimetric assay kits obtained from Funakoshi (Tokyo, Japan) and BioAssay Systems (Hayward, CA), respectively. Glycoalbumin (GA), a clinically relevant parameter for glycemic control in HD patients that is not influenced by either the presence of anemia or usage of an erythropoiesis-stimulating agent [9], in contrast to HbA1c, was measured as previously described [12].

### Statistical analysis

Values are shown as the mean ± standard deviation (SD) or median with interquartile range (IQR), depending on the presence or absence of a normal distribution. Comparisons of mean and median values between the T2DM and non-DM patients were performed using Student’s *t* test and Mann-Whitney’s *U* test, respectively. Correlations were assessed by Pearson’s correlation test or a nonparametric Spearman’s rank correlation test. Multiple regression analysis was performed to determine the independent associations of β-HB and arterial AcAc/β-HB ratio with albumin and uric acid, known to be relevant nutritional parameters for HD patients, after adjustments for age, gender, presence/absence of DM, and HD duration. Because of the skewed distribution of the values for these ketone bodies, they were entered into multiple regression analysis after logarithmic transformation. *P* values less than 0.05 were considered to indicate statistical significance. All calculations were performed using a Windows personal computer with the StatView V statistics software package (SAS Institute Inc., Cary, NC, USA).

## Results

### Clinical characteristics of HD patients just prior to starting HD session

The baseline clinical characteristics of the 49 enrolled HD patients (24 non-DM, 25 T2DM), determined just prior to a Monday or Tuesday morning HD session, 3 days after the previous session, are shown in Table 1. There were no significant differences for age, gender, HD duration, or inter-dialytic weight gain between the non-DM and T2DM groups, while BMI and albumin were significantly higher in the T2DM HD patients. Serum creatinine did not differ significantly between the groups. Fasting plasma glucose and GA, parameters for glycemic control, were significantly higher in the T2DM group. The underlying nephropathy type was diabetic nephropathy (*n* = 25), chronic glomerulonephritis (*n* = 10), nephrosclerosis (*n* = 9), polycystic kidney disease (*n* = 1), other disease (*n* = 3), and unknown disease (*n* = 1). All patients were free from significant acute illness or malignancy considered to have influence on metabolic status, as noted in our previous report [13]. Ten of the T2DM HD patients were not taking medication, while 3 were maintained on insulin combined with dipeptidyl peptidase-4 inhibitor and 12 were administered oral agents [dipeptidyl peptidase-4 inhibitor only (*n* = 9), dipeptidyl peptidase-4 inhibitor plus glinide (*n* = 1), dipeptidyl peptidase-4 inhibitor plus glinide plus α-glucosidase inhibitor (*n* = 2)].

**Table 1:** Baseline clinical characteristics of non-DM and T2DM HD patients before HD session

	All HD patients (*n* = 49)	Non-DM group (*n* = 24)	T2DM group (*n* = 25)	*P* value
Age, years	66.7 ± 11.5	68.6 ± 11.3	64.8 ± 11.6	0.2220
Gender, male/female	32/17	13/11	19/6	0.1085
BMI, kg/m^2^	22.7 ± 4.5	21.3 ± 4.3	24.0 ± 4.3	0.0494*
HD duration, years	4.5 (2.5–6.3)	4.7 (3.4–7.1)	4.3 (2.2–5.5)	0.1497
Interdialytic BW gain, %	5.3 (4.3–5.9)	5.4 (4.5–6.9)	5.2 (4.1–5.6)	0.2041
Serum urea nitrogen, mg/dL	60.5 ± 16.1	64.4 ± 18.3	56.8 ± 12.5	0.1096
Cre, mg/dL	10.0 ± 2.8	10.0 ± 2.7	10.1 ± 2.8	0.9045
Alb, g/dL	3.6 (3.4–3.8)	3.5 (3.2–3.7)	3.7 (3.5–3.9)	0.0141*
Fasting plasma glucose, mg/dL	122.0 (101.8–152.3)	115.5 (93.0–134.0)	145.0 (117.8–163.8)	0.0117*
Glycoalbumin, %	16.8 ± 3.0	15.2 ± 2.2	18.2 ± 3.0	0.0003*
CRP, mg/dL	0.11 (0.04–0.34)	0.11 (0.03–0.33)	0.11 (0.04–0.35)	0.6237
LDL-C, mg/dL	78.9 (56.3–102.0)	83.0 (58.5–107.0)	75.0 (51.8–95.8)	0.3127
Uric acid, mg/dL	5.9 (5.3–7.0)	6.2 (5.3–7.1)	5.8 (5.0–6.8)	0.3023
cCa, mg/dL	8.4 ± 0.6	8.5 ± 0.6	8.4 ± 0.6	0.6378
Pi, mg/dL	5.5 (4.9–6.1)	5.6 (4.9–6.1)	5.5 (4.8–6.2)	0.9920
Arterial blood				
pH	7.34 (7.32–7.36)	7.34 (7.31–7.36)	7.34 (7.33–7.36)	0.5754
HCO_3_, mEq/L	19.9 ± 2.2	19.4 ± 2.6	20.4 ± 1.7	0.2844
AcAc, μmol/L	26.0 (21.8–43.3)	26.0 (20.0–36.5)	28.0 (22.0–49.3)	0.3839
β-HB, μmol/L	20.0 (14.8–42.0)	17.0 (11.5–36.0)	29.0 (17.0–59.5)	0.0134*
AcAc/ β-HB ratio, μmol/μmol	1.18 (0.76–1.51)	1.35 (1.06–2.17)	0.91 (0.73–1.24)	0.0155*

Values in parentheses show range. *Significant difference between non-DM and T2DM groups (*p* < 0.05)

**Fig. 1:**
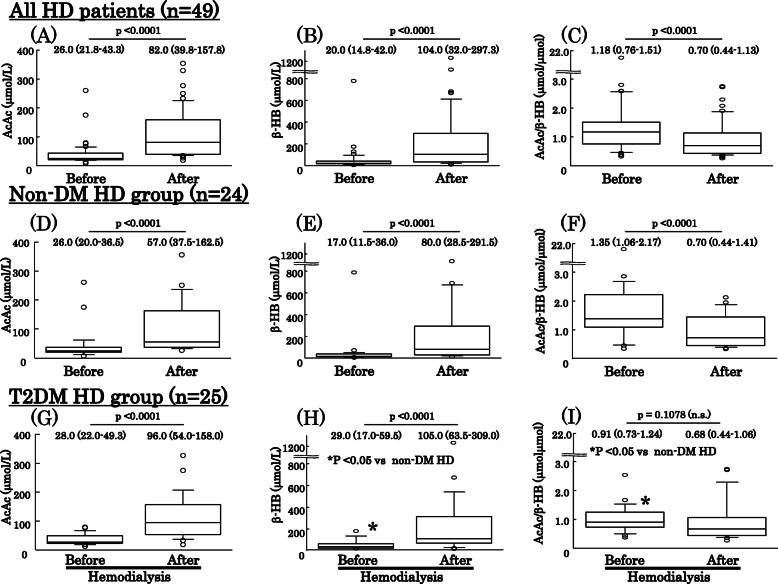
Changes in arterial AcAc, β-HB, and AcAc/β-HB ratio during 4-h hemodialysis session in all patients, as well as the non-DM, and T2DM groups. All HD patients, as well as after dividing into with or without T2DM, exhibited significant increases in arterial levels of AcAc and β-HB during a single 4-h HD session. Arterial AcAc/β-HB ratio was significantly reduced in all (**c**) and non-DM (**f**) patients, but not in the T2DM group (**i**). As a result, though the arterial AcAc/β-HB ratio was significantly higher in non-DM than T2DM HD patients before the HD session (*p* = 0.0134), it was not significantly different between those groups after the HD session (*p* = 0.1078)

As shown in Fig. 1, arterial β-HB was significantly higher in the T2DM HD [29.0 (range 17.0–59.5) μmol/L] as compared to the non-DM [17.0 (11.5–36.0) μmol/L] patients, whereas arterial AcAc was not significantly different between the groups [28.0 (22.0–49.3) vs. 26.0 (20.0–36.5) μmol/L], resulting in a significantly lower arterial AcAc/β-HB ratio in the T2DM HD [0.91 (0.73–1.24) μmol/L] than in the non-DM HD [1.35 (1.06–2.17) μmol/L] patients. An arterial AcAc/β-HB ratio < 1.0 was noted in 13 (56%) of the T2DM HD patients, which was significantly higher as compared to the non-DM group (*n* = 5, 20.8%) (*p* < 0.05, χ^2^ test). Neither arterial blood pH nor HCO_3_^−^ was significantly different between the groups.

### Regression analysis of correlation of arterial serum AcAc, β-HB, and arterial AcAc/β-HB ratio with various clinical parameters

Arterial AcAc, β-HB, and AcAc/β-HB ratio were examined for their correlation with various clinical parameters in all of the present cohort, as well as separately in the non-DM and T2DM groups (Table 2). Arterial AcAc was correlated with serum Pi in all as well as in the non-DM HD patients, and arterial β-HB was correlated with the rate of interdialytic weight gain, and urea nitrogen and uric acid in serum in all patients, and with fasting plasma glucose and LDL-C in the non-DM HD patients. Additionally, arterial AcAc/β-HB ratio was correlated with rate of interdialytic weight gain as well as urea nitrogen, creatinine, and uric acid in serum in all HD patients, and also with albumin, fasting plasma glucose, and LDL-C in the non-DM HD patients. Notably, fasting plasma glucose before the HD session was significantly correlated in a negative manner with β-HB and in a positive manner with AcAc/β-HB ratio in the non-DM, but not the T2DM group. The only significant correlations noted in the T2DM group was between arterial β-HB and rate of interdialytic weight gain, and between arterial AcAc/β-HB ratio and serum urea nitrogen and Pi.

**Table 2:** Correlations of arterial AcAc, β-HB, and AcAc/β-HB ratio in non-DM and T2DM HD patients

	All HD patients (*n* = 49)			Non-DM group (*n* = 24)			T2DM group (*n* = 25)		
	AcAc	β-HB	AcAc/β-HB	AcAc	β-HB	AcAc/β-HB	AcAc	β-HB	AcAc/β-HB
Age	0.092	0.035	−0.039	0.016	−0.118	0.063	0.219	0.358	−0.317
BMI	−0.023	− 0.068	0.160	− 0.024	− 0.214	0.268	− 0.014	− 0.180	0.327
HD duration	0.043	−0.162	0.190	−0.043	− 0.099	0.096	0.228	0.020	0.000
Interdialytic BW gain	−0.243	−0.472	0.360*	−0.228	− 0.453*	0.302	− 0.218	−0.420*	0.357
Serum urea nitrogen	−0.095	−0.350	0.434*	−0.020	− 0.414*	0.458*	− 0.130	−0.282	0.467*
Cre	−0.031	−0.267	0.343*	−0.078	− 0.373	0.425*	0.073	−0.108	0.254
Alb	0.140	0.021	0.112	−0.052	−0.352	0.442*	0.308	0.235	−0.030
Fasting plasma glucose	0.196	−0.013	0.207	0.143	−0.408*	0.592*	0.165	0.013	0.177
Glycoalbumin	0.088	0.166	−0.163	−0.038	− 0.338	0.342	− 0.002	0.230	− 0.318
LDL-C	0.054	0.193	−0.231	0.302	0.477*	−0.467*	−0.210	− 0.005	−0.137
Uric acid	0.057	−0.296	0.474*	0.083	−0.477*	0.664*	0.111	0.036	0.139
Pi	−0.338*	−0.242	0.204	−0.535*	− 0.109	−0.009	− 0.137	−0.304	0.445*

Correlation (r) was examined using Spearman’s Rank Correlation test

*Statistically significant difference (*p* < 0.05)

### Increased arterial ketone body levels during a single HD session

During a single session, all of the present HD patients exhibited significant increases in arterial levels of AcAc from 26.0 (range 20.0–36.5) μmol/L to 82.0 (39.8–157.8) μmol/L (*p* < 0.0001) (Fig. 1a) as well as β-HB from 20.0 (14.8–42.0) μmol/L to 104.0 (32.0–297.3) μmol/L (*p* < 0.0001) (Fig. 1b). Those increases resulted in a significant reduction in arterial AcAc/β-HB ratio from 1.18 (0.76–1.51) (μmol/μmol) to 0.70 (0.44–1.13) (μmol/μmol) (*p* < 0.0001) (Fig. 1c). When all HD patients were divided into T2DM and non-DM groups, the non-DM HD patients retained significant increases in arterial AcAc from 26.0 (20.0–36.5) μmol/L to 57.0 (37.5–162.5) μmol/L (*p* < 0.0001) (Fig. 1d) and β-HB from 17.0 (11.5–36.0) to 80.0 (28.5–291.5) μmol/L (*p* < 0.0001) (Fig. 1e). In T2DM HD patients, arterial AcAc and β-HB were significantly increased from 28.0 (22.0–49.3) μmol/L to 96.0 (54.0–158.0) μmol/L (*p* < 0.0001) Fig. 1g) and from 29.0 (17.0–59.5) μmol/L to 105.0 (63.5–309.0) μmol/L (*p* < 0.0001). Of interest, the reduction in arterial AcAc/β-HB ratio in the non-DM HD patients (Fig. 1f) during an HD session [from 1.35 (1.06–2.17) μmol/μmol to 0.70 (0.44–1.41) μmol/μmol] became statistically significant (p < 0.0001), while that change in T2DM HD patients (Fig. 1i) [from 0.91 (0.73–1.24) μmol/μmol to 0.68 (0.44–1.06) μmol/μmol] did not (*p* = 0.1078). As a result, though the arterial AcAc/β-HB ratio was significantly higher in the non-DM HD as compared to the T2DM HD patients before (*p* = 0.0134), it did not differ significantly between those groups after the HD session. Furthermore, arterial β-HB was found to be significantly lower in the non-DM HD patients before but not after the session. Together, these results suggest that the rate of increase in AcAc/β-HB ratio during the inter-dialytic period was higher in the non-DM HD patients, possibly due to a greater rate of β-HB reduction.

**Fig. 2:**
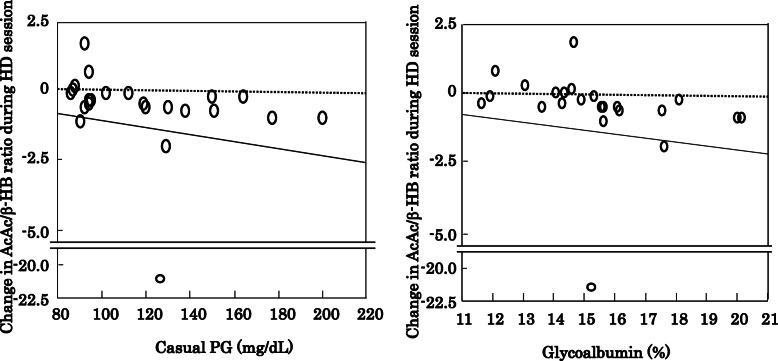
Correlation of change in AcAc/ β-HB ratio during HD session with fasting PG and glycoalbumin in non-DM HD patients. The change in AcAc/ β-HB ratio during the HD session was significantly and negatively correlated with fasting PG (**a**) (ρ = − 0.537, *p* = 0.0097) and glycoalbumin (**b**) (ρ = − 0.625, *p* = 0.0027) in the non-DM group

Changes in arterial blood pH and HCO_3_^−^ were not significantly correlated with those of arterial AcAc, β-HB, or AcAc/β-HB ratio in the full cohort, as well as after dividing into the T2DM and non-DM groups (data not shown). Notably, the baseline levels of both fasting plasma glucose and glycoalbumin before the HD session were significantly correlated in a negative manner with change in AcAc/β-HB ratio during the HD session in the non-DM HD patients (Fig. 2), but not in the T2DM HD patients (data not shown).

### Multiple regression analysis of β-HB and arterial AcAc/β-HB ratio with serum log albumin, Pi, and UA

Multiple regression analysis was performed to examine whether arterial β-HB or AcAc/β-HB ratio had a significant association with serum levels of albumin and uric acid in all of the present HD patients. When log β-HB was included as an independent variable, in addition to age, gender, presence/absence of T2DM, and log HD duration (Model 1), it emerged as a significant and independent factor showing an association with uric acid, but not albumin. When log β-HB was replaced with log AcAc/β-HB ratio (Model 2), that ratio showed a significant positive relationship with both albumin and uric acid. When log β-HB and log AcAc/β-HB ratio were simultaneously included as independent variables in Model 3, log AcAc/β-HB ratio, but not log β-HB, retained its independent and significant association in a positive manner with albumin and uric acid, indicating that arterial AcAc/β-HB ratio is superior to arterial β-HB as a clinically relevant marker for nutritional status in HD patients.

## Discussion

Results of the present study demonstrated an independent association of lower arterial AcAc/β-HB ratio, but not higher arterial β-HB, with reduced levels of albumin and uric acid in serum (Table 3). They indicate that lower arterial AcAc/β-HB ratio rather than higher arterial β-HB is a clinically relevant measurement for determining nutritional status in HD patients. Both lower serum albumin [14] and uric acid [15] have been established as has having an association with higher mortality in HD patients. Therefore, the present findings suggest that a lower arterial AcAc/β-HB ratio prior to starting an HD session may be an indicator of risk for malnutrition in HD patients and thus possibly increased mortality.

**Table 3:** Multivariate analysis to elucidate factors associated with arterial β-HB and AcAc/β-HB ratio in all HD patients (*n* = 49)

	Log albumin			Uric acid		
	Model 1	Model 2	Model 3	Model 1	Model 2	Model 3
Age	−0.172	−0.164	− 0.165	0.058	0.067	0.067
Gender (M/F, 1/2)	−0.0004	0.059	0.042	0.163	0.221	0.217
DM (−/+, 1/2)	0.313	0.386*	0.368*	−0.0004	0.073	0.068
Log (HD duration)	−0.256	−0.300*	− 0.319*	0.310*	0.237	0.232
Log β-HB	−0.092	–	0.244	−0.333*	–	0.061
Log AcAc/ β-HB ratio	–	0.300*	0.463*	–	0.533*	0.578*
p (R^2^)	0.0582 (0.214)	0.0128 (0.278)	0.0159 (0.299)	0.0404 (0.230)	0.0013 (0.362)	0.0029 (0.364)

*Statistically significant difference (*p* < 0.05)

An independent association of higher serum β-HB with greater number of cardiovascular events and all-cause mortality was recently shown in Japanese HD patients [4]. It is known that β-HB, an energy-rich short-chain (4-carbon) organic acid that can be freely diffused across the cell membrane, is capable of transporting energy to the heart and brain [16]. Furthermore, since β-HB is utilized as an energy source in the human heart in individuals either with or without T2DM [2], it has been suggested that a higher level of arterial β-HB should improve cardiac function by serving as a greater source of energy for the heart [17]. The present study showed that a reduction in arterial AcAc/β-HB ratio resulting from higher arterial β-HB, but not increased β-HB by itself, provides a better indicator for poor nutritional status in HD patients. β-HB is formed by reduction of AcAc in liver mitochondria by 3-hydroxybutyrate dehydrogenase, which requires oxidation of NADH to NAD^+^. That indicates that an arterial AcAc/β-HB ratio lowered to < 1.0 is a result of a highly reduced state of hepatic mitochondria (i.e., the NADH/NAD^+^ ratio) and reflects a reduced capacity of ATP synthesis within hepatic mitochondria [18]. It has also been shown that a normal arterial AcAc/β-HB ratio is usually > 1.0 [5], while a decrease to < 1.0 has been reported in various diseases associated with malnutrition, such as diabetic ketoacidosis, severe hypoxia, end-stage liver disease, hepatic ischemia, various metabolic disorders, and multiple organ failure [19], while another report showed it to be a factor related to increased risk of mortality [11]. Furthermore, it is also recognized as the metabolic basis for hepatocyte dysfunction [20] as well as lethal outcome in pediatric patients following heart surgery [11]. Additionally, recovery of arterial AcAc/β-HB ratio to > 1.0 was found to accompany normalization of graft metabolic function after liver transplantation [21].

In the present study, the arterial AcAc/β-HB ratio was significantly lower in our T2DM HD as compared to non-DM patients due to a significantly higher level of arterial β-HB (Table 1). Furthermore, because of the significant reduction in arterial AcAc/β-HB ratio during an HD session seen in the non-DM but not the T2DM group, we consider that the arterial AcAc/β-HB ratio is increased to a lesser degree in T2DM HD patients during the interdialytic period, mainly due to the smaller decrease in arterial β-HB in those patients. Diabetic ketoacidosis was previously reported to be associated with a decrease in arterial AcAc/β-HB ratio with a relatively high level of generation of β-HB, while insulin treatment decreases serum β-HB long before serum AcAc in diabetic ketoacidosis, resulting in an increased AcAc/ β-HB ratio [5]. Therefore, it is likely that insulin deficiency in T2DM HD patients is responsible for the higher β-HB level and lower AcAc/β-HB ratio, and possibly the unremarkable change in arterial AcAc/β-HB ratio noted during the interdialytic period, in contrast with non-DM HD patients. Indeed, the proportion of T2DM HD patients with an arterial AcAc/β-HB ratio ≤ 1.0 was significantly greater than that of non-DM HD patients in our study. Notably, the baseline level of fasting plasma glucose as well as glycoalbumin was significantly correlated in a negative manner with change in AcAc/β-HB ratio during the HD session only in the non-DM group (Fig. 2).

Increases in arterial AcAc and β-HB can be mainly explained by three mechanisms; (i) induction of alkalosis by dialysis with bicarbonate-containing dialysate, (ii) inhibition of insulin secretion by a reduced level of dialysate containing 125 mg/dL glucose, and (iii) stimulation of AcAc production by dialysis with acetate-containing dialysate. Therefore, the reduction in plasma glucose during the HD session was greater in the present non-DM HD patients with worse glycemic control. Since the non-DM group was shown to retain insulin secretory capacity, those with worse glycemic control who are exposed to a greater suppressive effect of insulin secretion during an HD session might have a higher AcAc/β-HB ratio due to a larger increase in β-HB. In contrast, it is possible that in T2DM HD patients with severely impaired insulin secretor capacity, worse glycemic control did not have an effect to a large enough degree to inhibit insulin secretion during the HD session.

It has been shown that alkalosis stimulates lipolysis [22] to produce free fatty acids with a resultant increase in ketone production [23]. However, the absence of a correlation of arterial blood pH or bicarbonate either at the baseline or change during the HD session with arterial ketone bodies might negate the involvement of alkalotic change in increased arterial ketone bodies during a session. Although it has been reported that a high concentration of acetate in dialysate increases serum AcAc and β-HB after an HD session [24, 25], we found that changes in AcAc and β-HB levels in the present HD patients were not significantly different between those treated with acetate-free Carbostar dialysate and 8.0 mM of acetate-containing Kindaly 4E dialysate (personal observation, data not shown), clearly demonstrating that 8.0 mM of acetate in the dialysate did not contribute to an increase in arterial ketone bodies during the HD session.

As demonstrated in the present multivariate analysis (Table 3), even after inclusion of presence/absence of DM as an independent variable, arterial β-HB and arterial AcAc/β-HB ratio both retained a significant association with serum albumin and uric acid, markers for nutrition and mortality in HD patients [14, 15]. Those results support the notion that lower arterial AcAc/β-HB ratio is a clinically relevant marker of poor nutritional status in HD patients.

This study has some limitations. First, the sample size was small and all subjects had Japanese ethnicity. On the other hand, strong points include performance by a single institution and dialysis performed with one dialysate under the same situation managed by the same staff.

## Conclusions

The present results indicate that the lower arterial AcAc/β-HB ratio seen in DM HD patients is mainly due to higher arterial β-HB level. That lower ratio was also shown to be significantly associated with markers of poor nutritional status, such as reduced levels of serum albumin and uric acid, suggesting that a low arterial AcAc/β-HB ratio is related to greater mortality in those patients.

## Data Availability

Datasets used and/or analysed for the current study are available from the corresponding author upon reasonable request.

## Abbreviations

AcAc: Acetoacetate
Alb: Albumin
ATP: Adenosine triphosphate
β-HB: β –hydroxybutyrate
BMI: Body mass index
Bw: Body weight
Ca: Calcium
Cre: Creatinine
CRP: C-reactive protein
CVD: Cardiovascular disease
T2DM: Type 2 diabetes mellitus
GA: Glycoalbumin
HD: Hemodialysis
IQR: Interquartile range
LDL-C: Low density lipoprotein cholesterol
NAD: Nicotinamide adenine dinucleotide
PG: Plasma glucose
Pi: Phosphate
SD: Standard deviation
